# Editorial: Advancement of chemotherapy in breast cancer: predictive markers, resistance mechanism and therapeutic strategies

**DOI:** 10.3389/fonc.2026.1896583

**Published:** 2026-07-03

**Authors:** Richard Beatson, Jinhai Deng, Teng Pan, Zhendong Shi, Ming Zhao

**Affiliations:** 1 School of Cancer and Pharmaceutical Sciences, King’s College London, London, United Kingdom; 2 Guangzhou Baiyunshan Pharmaceutical Holding Co., Ltd. Baiyunshan Pharmaceutical General Factory, Guangdong Province Key Laboratory for Core Technology of Chemical Raw Materials and Pharmaceutical Formulations, Guangzhou, China; 3 Maternity and Child Healthcare Hospital of Longgang District, Shenzhen City (Longgang Maternity and Child Institute of Shantou University Medical College, Shantou, China; 4 Tianjin Medical University Cancer Institute and Hospital, Tianjin, China; 5 University of Kansas, Lawrence, KS, United States

**Keywords:** biomarkers, breast cancer, chemotherapy, resistance, therapies

## Introduction

As editors of this Research Topic, it was our pleasure to review a wide range of fascinating articles in the field of chemotherapy in breast cancer. We particularly focused on predictive markers, resistance mechanisms and therapeutic strategies to showcase three growth areas, resulting in 16 accepted articles: 10 original research, 1 hypothesis and theory, 2 review, 1 systematic review, 2 clinical trial. In this editorial we summarise the main findings of each.

## Trials

Dewidar et al. reported on an open-label clinical trial (NCT06186700) evaluating pentoxifylline (PTX) in 106 breast cancer patients undergoing AC/T chemotherapy, comparing standard chemotherapy alone or combined with PTX. Results indicated that PTX significantly reduced the incidence of grade 2 or higher peripheral sensory neuropathy compared to control (75% vs 90.7%) and delayed its onset. Furthermore, PTX lowered grade 2 or higher mucositis incidence during both AC and taxane phases without significant differences in hematological, cardiac, renal, or hepatic toxicities. In conclusion, co-administering PTX significantly decreases and postpones chemotherapy-induced neuropathy and mucositis.

A randomized, placebo-controlled trial (NCT06459193) reported by Iqbal et al. found that 35 mg daily trimetazidine significantly reduces the incidence of grade 2–3 paclitaxel-induced peripheral neuropathy in non-metastatic breast cancer patients compared to placebo. Treatment with trimetazidine notably decreased paresthesia, peripheral motor neuropathy, and dysesthesia while improving neurotoxicity-related quality of life and increasing serum nerve growth factor levels. Trimetazidine was shown to be a safe, effective option for mitigating early neuropathy, though further large-scale studies are warranted.

## Hypothesis and theory

Li et al. explore the clinical implications of targeting lipid metabolism reprogramming in breast cancer, which drives progression, drug resistance, and poor prognosis. The authors highlight key enzymes and therapeutic targets and examine relevant clinical trials, concluding that there is an urgent need for predictive biomarkers to identify responsive patient subpopulations. To address this, the authors propose the Selective Lipid Metabolism Therapy Benefit Hypothesis, emphasizing the necessity of personalized medicine to optimize these targeted therapies for individual breast cancer patients.

## Original articles

A genomic and bioinformatics study by Zhang et al. investigated the role of ATP-induced cell death (AICD) in breast cancer (BC) using TCGA data. The researchers identified an 18-gene AICD signature and constructed an accurate prognostic model. High-risk populations demonstrated significantly lower overall survival rates, while low-risk groups exhibited elevated anti-tumour immune cell and immune checkpoint molecule expression. Experimentally, increased ATP concentrations reduced BC cell viability, inhibited migration, promoted apoptosis, and altered the expression of CLIC6, SLC1A1, CEMIP and ANO6. Ultimately, inducing AICD may protect against BC progression, providing an option for personalized treatment strategies.

Using 20 years’ worth of data from 21,058 female participants, Li et al.‘s study evaluated the Systemic Immune-Inflammation Index (SII) as a predictor for breast cancer incidence and mortality. Results revealed a non-linear relationship for both outcomes. Breast cancer incidence showed an inverse L-shaped association; below an SII/100 threshold of 5.09, risk significantly increased. Conversely, all-cause mortality among the 557 breast cancer patients exhibited a J-shaped relationship; above a threshold of 5.22, mortality risk escalated significantly. As such, SII may well prove to be a valuable prognostic tool associating with breast cancer development and patient survival.

Liao et al. analyzed 248 triple-negative breast cancer (TNBC) patients undergoing neoadjuvant chemotherapy (NAC) across two centers to identify efficacy and prognostic predictors. Multivariable analysis showed that high biopsy-sTILs, biopsy-Ki67 >20%, and positive biopsy-AR predicted a pathological complete response (pCR). Key predictors for disease-free and overall survival included ypN status, postoperative sTILs, radiotherapy, and NAC effectiveness. The researchers developed a predictive nomogram model, which demonstrated high discrimination and accuracy. C-indices ranged from 0.729 to 0.816 for pCR, 0.865 to 0.895 for DFS, and 0.860 to 0.899 for OS, offering a robust prognostic tool for clinical use.

A real-world retrospective study by Tang et al. evaluated therapeutic drug monitoring (TDM) of docetaxel in 180 breast cancer patients with the aim of improving the management of adverse reactions. Standard dosing based on body surface area ignores wide metabolic variations among individuals. While patient demographics showed no significant differences, univariate analysis linked specific docetaxel area under the curve (AUC) levels to variations in albumin, creatinine, nausea, vomiting, rashes, and alopecia. Binary logistic regression confirmed that an AUC value exceeding 2.5 mg·h/L significantly escalates the risk of rashes, alopecia, and altered renal/hepatic markers. Consequently, docetaxel TDM helps optimize clinical dosing and minimize toxicity.

In a retrospective, propensity-matched cohort study Neiman et al. evaluated whether dexrazoxane (DZR) mitigates doxorubicin (DOX) cardiotoxicity in 152 cancer patients. Cardiotoxicity was measured by changes in ejection fraction (EF), while additional toxicities were graded via CTCAE. Results showed no statistically significant difference in median EF change between the DOX alone group and the DOX + DZR group (-2% vs -0.7%, p = 0.9174), suggesting a lack of immediate cardioprotective benefit. However, the DOX + DZR group experienced significantly higher rates of grade 4 anemia (p = 0.0002) and grade 4 neutropenia (p = 0.0013). Thus, DZR co-administration may escalate hematologic risks without immediate cardiac advantages.

Shi et al. assessed the diagnostic performance of breast MRI in 554 female patients following neoadjuvant therapy (NAT). While the overall accuracy of MRI in evaluating treatment response was 77.44%, multivariable analysis revealed that ER-negative status, the absence of ductal carcinoma *in situ* (DCIS), and the coexistence of mass lesions with non-mass enhancement, significantly reduced this accuracy. By integrating independent clinicopathological and MRI predictors, the researchers constructed a nomogram to predict pathological complete response. The model demonstrated robust discrimination, achieving an AUC of 0.894 in the training cohort and 0.888 in the validation cohort.

Song et al. investigated the role of the *KNSTRN* gene in triple-negative breast cancer (TNBC) using RNA-seq, single-cell transcriptomics, and *in vitro* validation. The analysis revealed that *KNSTRN* is significantly overexpressed in TNBC compared to normal tissues and other breast cancer subtypes, correlating directly with poor patient prognosis. Functional enrichment and immune analyses linked high *KNSTRN* expression to cell cycle promotion and reduced CD8+ T-cell infiltration. Knocking down *KNSTRN* in cell lines successfully inhibited cancer proliferation and migration. Ultimately, *KNSTRN* represents a promising prognostic biomarker and a potential therapeutic target for reversing the immunosuppressive TNBC microenvironment.

Gao et al. established patient-derived primary cancer cell lines from four HR+/HER2− breast cancer patients to investigate docetaxel resistance mechanisms. RNA sequencing and protein-protein interaction network analysis identified the *COL1A2* gene as significantly overexpressed in docetaxel-resistant cells. Clinically, elevated *COL1A2* expression negatively correlated with pathological complete response rates and recurrence-free survival in patients undergoing neoadjuvant AC-T chemotherapy. Functional assays confirmed that higher *COL1A2* expression directly reduces docetaxel sensitivity in breast cancer cells. As such, *COL1A2* serves as a promising biomarker to predict taxane resistance and guide personalized neoadjuvant chemotherapy selection for HR+/HER2− breast cancer.

A retrospective study by Cai et al. analyzed 767 premenopausal patients with triple-negative breast cancer (TNBC) to investigate chemosensitivity and intrinsic resistance following surgery and standard chemotherapy. Multivariable analysis identified lower stromal tumor-infiltrating lymphocyte (sTIL) expression, HER2 IHC 2+/FISH-negative status, advanced T-stage, and higher N-stage as independent predictors of poorer disease-free survival (DFS). The researchers integrated these four variables into a prognostic nomogram, which demonstrated excellent predictive accuracy (C-index: 0.862 training, 0.861 validation). Ultimately, an immunosuppressive microenvironment was shown to drive chemotherapy resistance, offering a biomarker-informed framework to stratify patients for immunotherapy or antibody-drug conjugates.

Long et al. evaluated the clinical utility of the albumin-bilirubin (ALBI) score and age in predicting trastuzumab resistance in 95 HER-2 positive breast cancer patients. Among the cohort, 11 patients (11.6%) developed drug resistance. Multivariable logistic regression identified advanced age, a higher ALBI score, and elevated CEA levels as significantly associated with resistance. Both the ALBI score and age emerged as independent predictors. ROC curve analysis demonstrated that combining age and ALBI score provided superior predictive performance, yielding an AUC of 0.771 with 72.7% sensitivity and 77.4% specificity. Consequently, this combined metric facilitates early identification of trastuzumab resistance risk.

## Reviews

Li et al. explore how eukaryotic initiation factor 3b (eIF3b) drives Adriamycin (ADM) resistance in breast cancer by regulating protective autophagy and interacting with the Wnt/β-catenin pathway. The protein eIF3b is overexpressed in ADM-resistant tissues and cell lines, while its downregulation suppresses autophagy and restores ADM sensitivity. Synthesizing these mechanisms, this review highlights eIF3b as an upstream autophagy regulator that promotes multidrug resistance. By proposing interaction models within the eIF3b-autophagy-Wnt/β-catenin network, the authors identify this axis as a promising therapeutic target to reverse chemoresistance and improve treatment outcomes for patients.

In their review, Wei et al. highlight oral selective estrogen receptor degraders (SERDs)—including elacestrant, camizestrant, giredestrant, and imlunestrant—for treating advanced hormone receptor-positive (HR+) breast cancer. Endocrine therapy resistance, driven by *ESR1* mutations, presents a substantial clinical challenge. Next-generation oral SERDs overcome this by offering superior bioavailability, robust estrogen receptor degradation, and manageable toxicity profiles. Combining these agents with CDK4/6 or mTOR inhibitors further improves outcomes in treatment-resistant populations. By evaluating recent clinical trial data and pharmacological profiles, this review offers insights into optimizing treatment sequencing and advancing precision medicine strategies to overcome resistance in advanced HR+ disease.

## Systemic review

A systematic review by Alotaibi evaluated 72 studies following PRISMA guidelines to examine recent advancements in epigenetic research for breast cancer (BC). It focuses on DNA methylation, histone modification, and non-coding RNAs as potential diagnostic and prognostic biomarkers for early detection and tracking disease progression. The authors highlight the critical role of abnormal epigenetic changes in driving cancer initiation, metastasis, and drug resistance. The review details the therapeutic promise of epigenetic drugs (epi-drugs), such as DNMT and HDAC inhibitors, alongside nanotechnology-assisted delivery systems to minimize toxicity. Ultimately, integrating epigenetic insights offers innovative pathways for personalized, targeted BC management.

## Conclusion

This comprehensive body of work underscores a crucial paradigm shift toward biomarker-driven, personalized breast cancer management. Clinical trials demonstrate successful toxicity mitigation using pentoxifylline and trimetazidine for chemotherapy-induced neuropathy. Concurrently, advanced prognostic nomograms, therapeutic drug monitoring, and genomic signatures—such as the Systemic Immune-Inflammation Index, *KNSTRN*, *COL1A2*, and sTIL/HER2-low profiles—offer robust frameworks to predict survival, pathological complete responses, and taxane or trastuzumab resistance. Furthermore, critical reviews on eIF3b-mediated autophagy, next-generation oral SERDs, and epigenetic modifications map out novel therapeutic targets. Together, these studies bridge molecular insights and clinical applications to optimize precision oncology, overcome resistance, and improve patient outcomes.

